# The Role of Ultrasound in Postoperative Assessment of Biceps Tenodesis: A Case Report

**DOI:** 10.7759/cureus.86099

**Published:** 2025-06-15

**Authors:** Mohammed Hamzah Hameed, Mainul Huda, Maahi Qureshi, Areefa Momtaz, Salman F Hameed

**Affiliations:** 1 Emergency Medicine, Sandwell and West Birmingham NHS Trust, Birmingham, GBR; 2 Acute Medicine, Sandwell and West Birmingham NHS Trust, Birmingham, GBR; 3 Geriatrics, Sandwell and West Birmingham NHS Trust, Birmingham, GBR; 4 Orthopedics, Zia Medical Center, Dubai, ARE

**Keywords:** biceps tenodesis, mri, msk ultrasound, musculoskeletal radiology, post-op rehabilitation, slap tear, sports surgery

## Abstract

Superior labral anterior-posterior (SLAP) tears frequently occur following overhead activities or traumatic shoulder injuries. While magnetic resonance arthrography (MRA) remains the gold standard for diagnosis, ultrasound shows promise for postoperative monitoring of long head biceps tendon (LHBT) tenodesis. We present the case of a 55-year-old man with a type III SLAP tear who developed a screw cut-out after subpectoral tenodesis. Initial conservative management was unsuccessful, necessitating surgical intervention. At one-year follow-up, ultrasound evaluation demonstrated an empty bicipital groove, preserved tendon architecture, and stable subpectoral fixation, findings later confirmed by magnetic resonance imaging (MRI).

The ultrasound findings were largely concordant with the MRI findings while offering the advantages of real-time dynamic assessment, cost-effectiveness, and immediate bedside availability. This case demonstrates the reliability of ultrasound for postoperative monitoring of biceps tenodesis. The modality enables early detection of complications without radiation exposure, though operator experience remains important for optimal results. While MRI retains superiority for preoperative diagnosis, ultrasound serves as an effective screening and follow-up tool with the unique capability to assess tendon motion dynamically. However, its accuracy is operator-dependent, and variability in interpretation between clinicians can affect diagnostic consistency, factors that are particularly important to consider in this context.

The findings of this case support the growing role of ultrasound in sports medicine practice, bridging the gap between clinical examination and advanced imaging. Further standardization of ultrasound protocols could significantly improve postoperative shoulder assessment. The case highlights how ultrasound can optimize patient care pathways while maintaining diagnostic accuracy for biceps tenodesis monitoring.

## Introduction

Superior labral anterior-posterior (SLAP) tears represent a spectrum of injuries to the superior glenoid labrum, where the long head of the biceps tendon (LHBT) anchors to the glenoid rim. The LHBT not only stabilizes the glenohumeral joint but also contributes to elbow flexion and forearm supination. SLAP tears are a common cause of shoulder pain, particularly in individuals engaged in overhead activities or those who have sustained traumatic shoulder injuries. The Snyder classification system categorizes SLAP tears into four distinct types based on their morphological characteristics, with Maffet et al. later expanding this classification with three additional variants [1,2]. While type I lesions typically respond to conservative management, type II tears generally require surgical repair. Complex type III and IV lesions often necessitate either LHBT tenotomy or tenodesis, with the latter being preferred for young, active patients due to superior functional outcomes [3,4]. 

Accurate clinical diagnosis of SLAP tears remains clinically challenging, as physical examination tests vary widely in sensitivity and specificity. Commonly employed maneuvers include O'Brien’s active compression test (sensitivity 63%-94%, specificity 28%-73%) and Speed’s test (sensitivity 32%-60%, specificity 38%-79%). The variability in these values underscores the importance of combining multiple tests and correlating findings with imaging studies to improve diagnostic accuracy. [5]. 

Plain radiographs are generally unremarkable in LHBT pathologies. Magnetic resonance arthrography (MRA) currently serves as the gold standard, demonstrating 78%-84% sensitivity and 95%-99% specificity for SLAP tears [6]. However, it presents logistical challenges in resource-limited settings due to high costs, limited availability, and lengthy scan times. Additionally, contraindications (e.g., metallic implants, pacemakers, cochlear implants, in addition to claustrophobia, and morbid obesity) and the need for specialized infrastructure further restrict its accessibility. Given these limitations, there is growing interest in exploring alternative imaging modalities such as ultrasound. The role of ultrasound in diagnosing SLAP tears is debatable; it is not considered a primary diagnostic tool for SLAP lesions, and for that, MRI remains the gold standard for diagnosis. Ultrasound, however, shows promise as a follow-up modality, particularly for monitoring healing or post-intervention progress [7]. 

An uncommon but clinically significant complication of LHBT tenodesis is interference screw fixation failure, which typically manifests within the two-month postoperative window [8]. An interference screw is a specialized orthopedic implant used to secure soft tissue grafts, such as tendons or ligaments within bone tunnels during surgical procedures like biceps tenodesis, ACL reconstruction, or rotator cuff repair. It provides strong fixation allowing for tendon healing and early mobilization. This hardware-related failure may result from technical factors, excessive loading, or suboptimal bone quality at the tenodesis site [9]. While MRI remains the diagnostic gold standard for detecting postoperative complications, its aforementioned limitations can restrict routine use for serial monitoring. 

In contrast, ultrasound offers distinct advantages, including real-time dynamic evaluation, the absence of metallic artifacts, and the ability to perform serial assessments, which enables early detection of implant migration or tendon abnormalities without the delays associated with MRI. When performed by experienced operators, ultrasound demonstrates comparable accuracy to MRI in evaluating tendon integrity and hardware position [10,11]. 

We present the case of a 55-year-old man with a type III SLAP tear who underwent subpectoral LHBT tenodesis using an interference screw. Postoperatively, the patient experienced a rare complication related to screw cut-out, which was successfully managed with surgical intervention. This case demonstrates the clinical utility of ultrasound as an effective postoperative imaging modality for biceps tenodesis assessment, offering comparable diagnostic accuracy to MRI while addressing practical limitations of cost, accessibility, and real-time dynamic evaluation. 

## Case presentation

**Figure 1:**
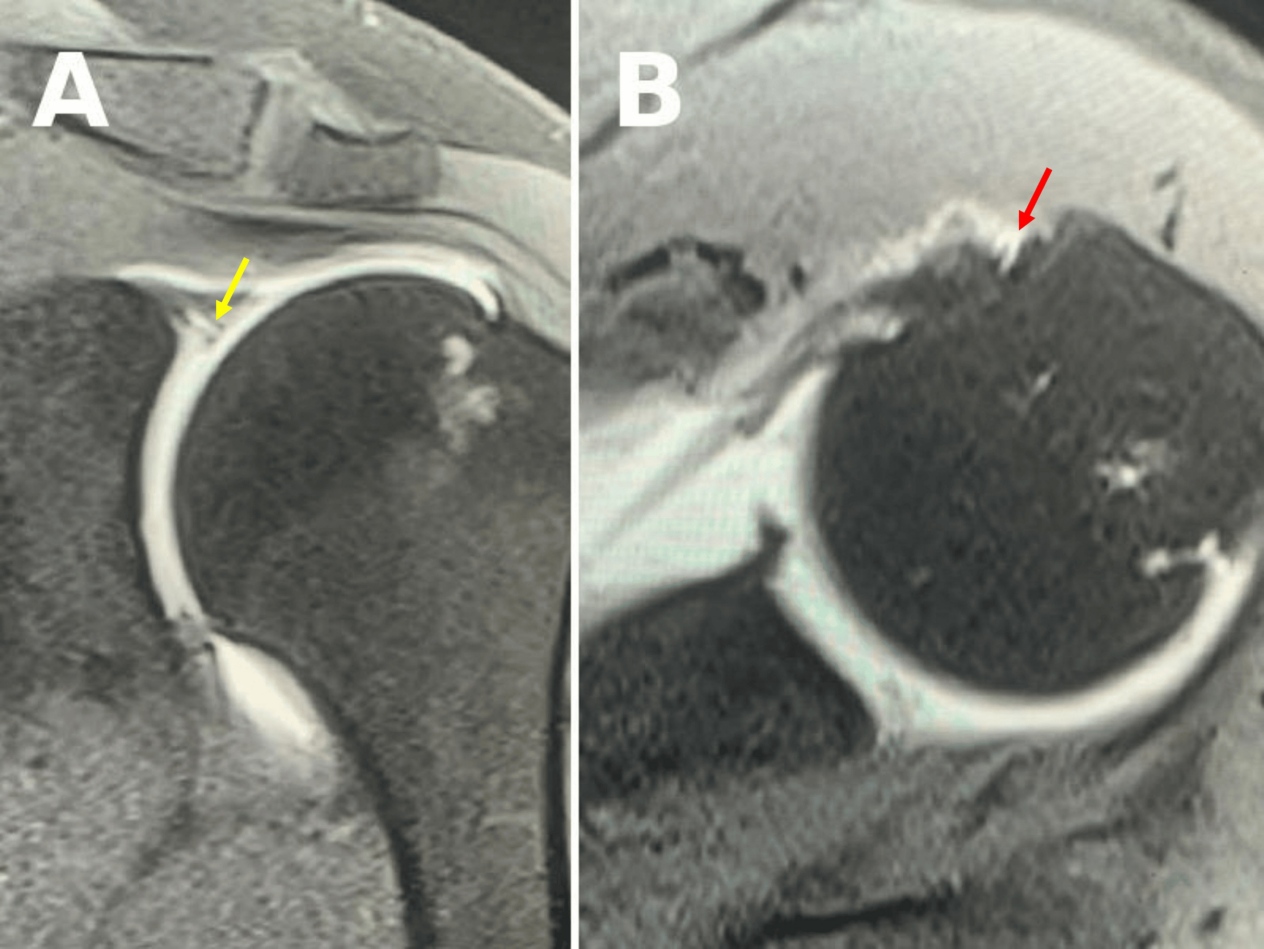
Preoperative MRA with contrast of the left shoulder showing a SLAP tear. (A) T2-weighted coronal view with a visible partial tear of the biceps (yellow arrow). (B) T2-weighted axial view showing contrast communicating with synovial bicipital groove (red arrow). MRA: magnetic resonance arthrogram, SLAP: superior labral anterior-posterior.

A 55-year-old right-hand-dominant man presented with anterior and lateral left shoulder pain for six months following a gym-related traction injury. The patient reported persistent pain and clicking in the left shoulder, which worsened with overhead activities. Clinical examination revealed positive Neer's, Speed's, and Yergason's tests, suggestive of subacromial impingement and biceps tendon pathology [12]. Plain radiographs demonstrated a type III hooked acromion, and further evaluation with MRA (Figure 1) confirmed a type III SLAP tear. 

Initial conservative management, including physiotherapy, anti-inflammatory medications, and corticosteroid injections into the subacromial space and biceps sheath, failed to provide relief. Following six months of conservative management, the patient underwent left shoulder arthroscopy, anterior acromioplasty, and mini-open subpectoral LHBT tenodesis using an 8 mm × 12 mm polyether ether ketone (PEEK) interference screw. The preoperative assessment established good bone health with minimal risk factors for osteoporosis. The subpectoral approach was selected over the suprapectoral technique based on the surgeon's experience and to minimize postoperative pain. The immediate postoperative period was uneventful.

**Figure 2:**
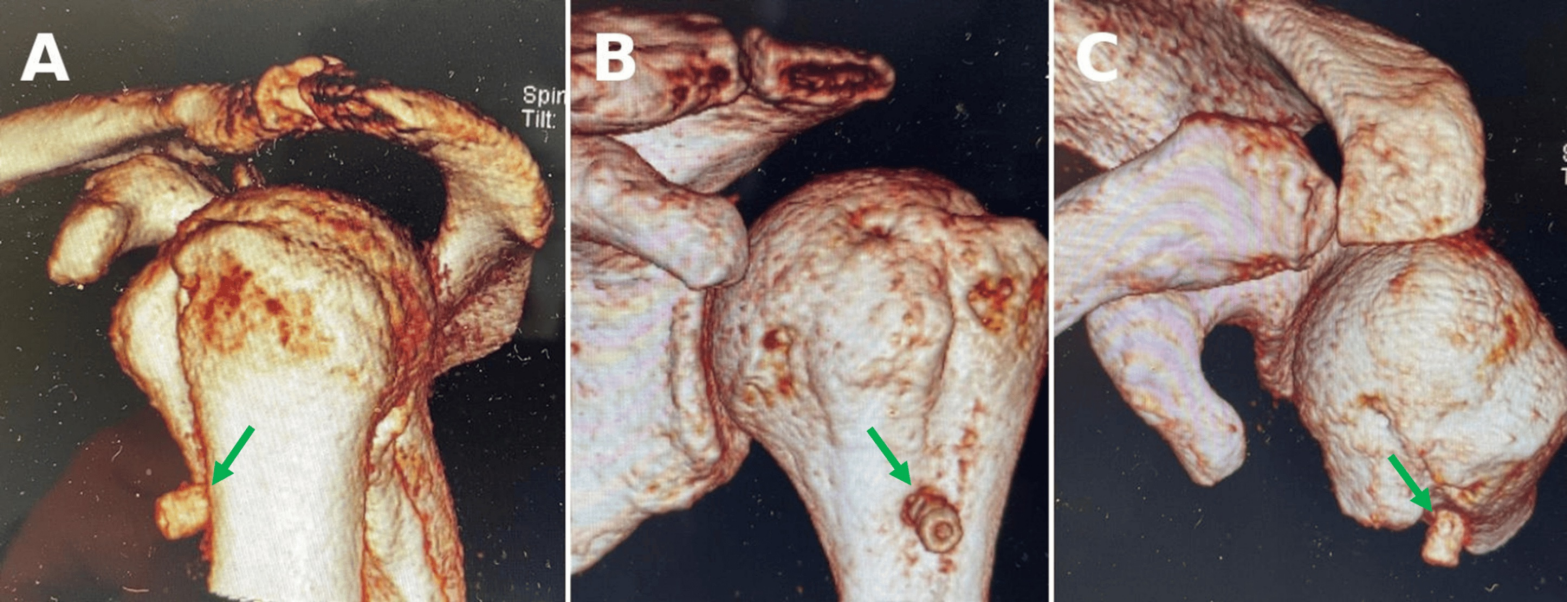
Eight weeks postoperative CT of the left shoulder (three-dimensional reconstruction) demonstrating cut-out screw position in different views. (A-C) Visible screw penetration (green arrows). CT: computed tomography.

**Figure 3:**
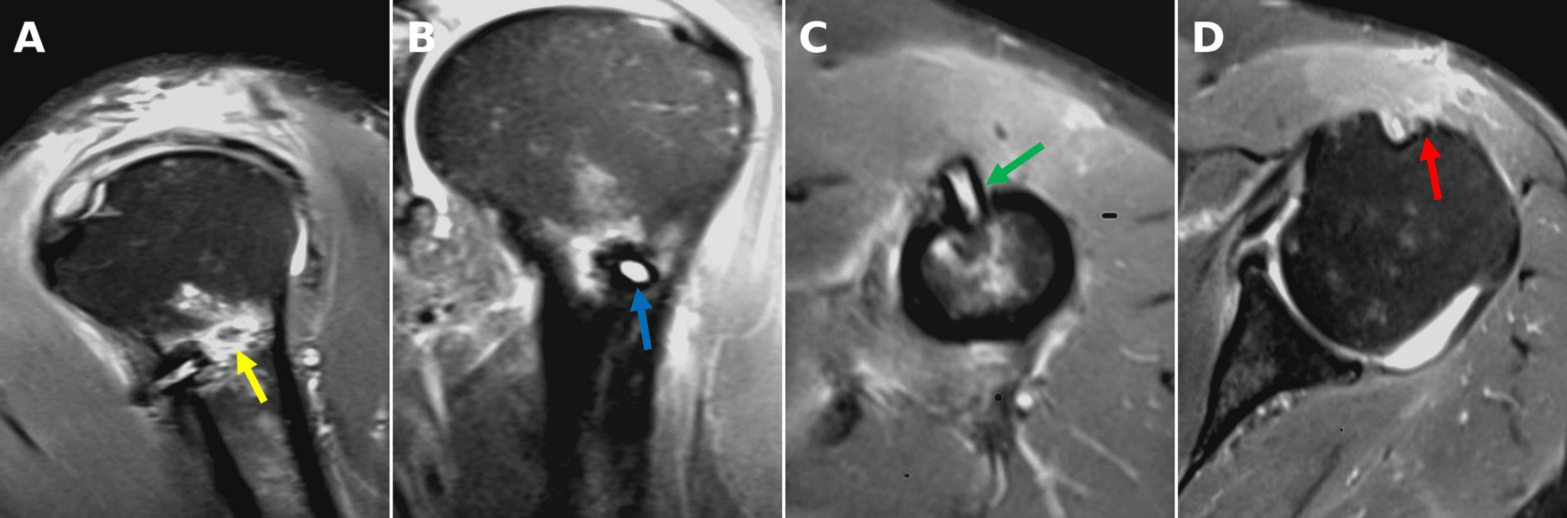
Eight weeks postoperative T2-weighted MRI of the left shoulder. (A) Screw cut-out with intraosseous inflammation (yellow arrow). (B) Sagittal view of screw penetration through proximal humerus (blue arrow). (C) Axial view of screw penetration through proximal humerus (green arrow). (D) Empty bicipital groove (red arrow). MRI: magnetic resonance imaging.

However, during postoperative physiotherapy at six weeks, the patient experienced severe anterior shoulder pain. The patient reported strict adherence to a tailored physiotherapy plan and denied any history of over-exertion. At eight weeks, computed tomography (CT) (Figure 2) and MRI revealed a screw cut-out with impingement on the anterior deltoid (Figure 3). 

The patient underwent open surgical removal of the displaced interference screw without replacement. The intraoperative assessment revealed localized inflammation surrounding the hardware; fortunately, the biceps tendon demonstrated partial incorporation within the bicipital groove and remained stable without evidence of pullout. Given these findings, along with secure fixation provided by mature scar tissue within the pre-existing bone tunnel, no additional screw fixation was required. This selective hardware removal successfully addressed the mechanical impingement while preserving tenodesis integrity. 

Follow-up at six months demonstrated a full range of motion of the shoulder with a visual analog scale (VAS) score of 2. At 12-month follow-up, the VAS score was 0, and the patient could perform gym workouts with relatively moderate weight. During the same time, a comprehensive ultrasound evaluation was conducted by a musculoskeletal radiology consultant (>20 years of experience in ultrasound) to assess the post-tenodesis biceps tendon using a standardized protocol. With the patient seated and the examiner positioned posteriorly, a high-resolution linear transducer (6-14 MHz) was used with optimized shoulder imaging parameters (i.e., frequency, depth, and gain) to optimize image quality based on the structures being visualized. Systematic evaluation included dynamic assessment in both longitudinal and transverse planes, with representative images digitally archived for subsequent analysis. 

**Figure 4:**
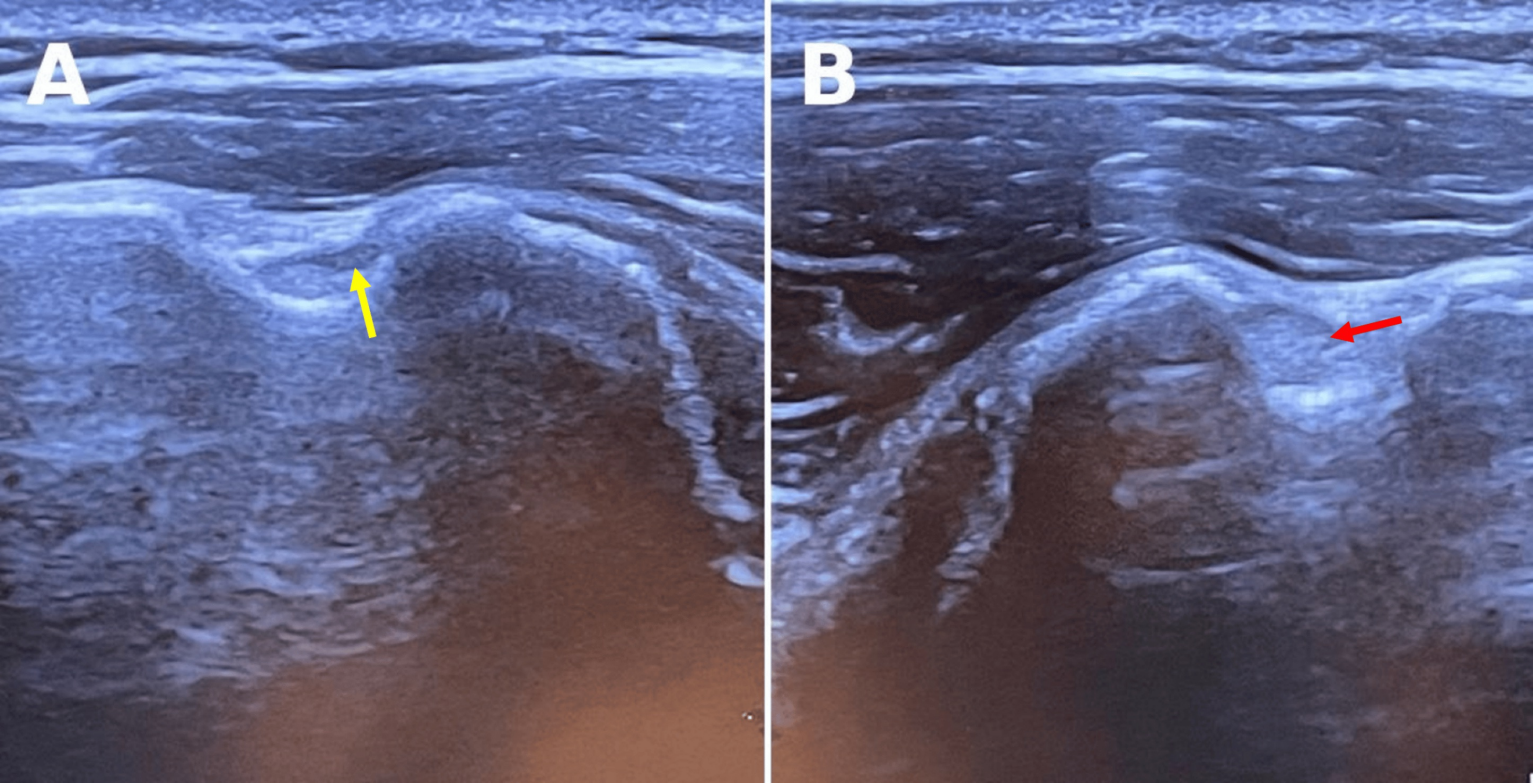
Comparative 12-month postoperative ultrasound transverse view of the left and right shoulders. (A) Transverse view of the left shoulder displaying absent LHBT in the bicipital groove (yellow arrow). A hyperechoic posterior wall with acoustic enhancement is noted below the bicipital groove. (B) Transverse view of the right shoulder with LHBT present in the bicipital groove (red arrow). LHBT: long head biceps tendon.

**Figure 5:**
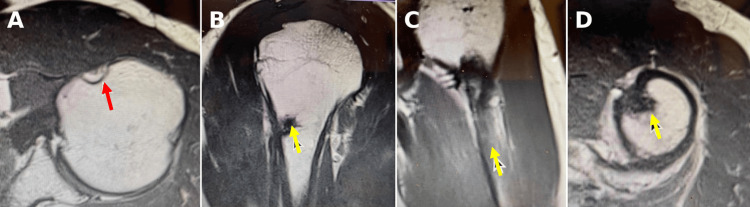
Twelve-month postoperative T1-weighted MRI of the left shoulder. (A) Absent LHBT in the bicipital groove (red arrow). (B-D) Subpectoral biceps tendon in the intraosseous tunnel (yellow arrows). MRI: magnetic resonance imaging, LHBT: long head biceps tendon.

Ultrasound revealed definitive evidence of successful tenodesis, including an empty bicipital groove, preserved tendon fibrillation, and stable subpectoral fixation (Figure 4), all subsequently verified by MRI (Figure 5). This correlation underscores ultrasound's validity for postoperative assessment following biceps tenodesis procedures. 

## Discussion

The role of ultrasound in the direct diagnosis of intra-articular SLAP lesions remains limited. MRI remains the reference standard for the diagnosis of SLAP tears, with current literature reporting ultrasound sensitivities ranging from 72% to 89% when compared to MRI [7,13]. Nonetheless, when performed by experienced operators using standardized protocols, ultrasound offers distinct advantages as a screening and follow-up modality, particularly in resource-constrained settings where infrastructure barriers, patient contraindications, or logistical constraints limit MRI access. Furthermore, ultrasound offers the unique advantage of real-time dynamic assessment, enabling evaluation of the tendon across a range of shoulder and elbow movements. Ultrasound has notable limitations as well, including difficulty in evaluating deep intra-articular structures and reduced image quality in patients with significant body habitus, which can hinder diagnostic accuracy. The advantages then must be weighed against inherent operator dependence, inter-observer variability in image interpretation, and patient anatomical factors, which may affect diagnostic reliability. 

The management of SLAP tears is guided by its classification, with surgical intervention typically indicated for type III/IV lesions. Among surgical options, LHBT tenodesis has emerged as the preferred approach for young, active patients [4]. Postoperative surveillance remains critical, as hardware-related complications like screw cut-outs (reported in 3%-8% of cases) require prompt diagnosis [8]. While MRI remains the gold standard imaging for detecting such complications, ultrasound demonstrates significant potential as a first-line surveillance tool, offering comparable accuracy (88%-92% sensitivity for screw malposition) with advantages of serial dynamic assessment, cost-effectiveness, and immediate bedside availability. 

In the case of our patient, ultrasound was demonstrated to be a reliable imaging modality for postoperative assessment, offering similar findings to MRI. Although our patient ultimately required MRI for definitive diagnosis of screw cut-out, ultrasound might help reduce the need for MRI in some stable postoperative cases, in a resource-limited setting, by providing effective ongoing monitoring. It is important to note that these findings come from a single case and may not apply to all patients. Further research involving a larger patient cohort with different surgical techniques and a variety of body types is needed to better understand the role of ultrasound in this setting. Ultrasound’s cost-effectiveness, widespread availability, and capacity for real-time dynamic assessment of both tendon integrity and implant position make it particularly valuable for serial postoperative evaluations [10,11]. The modality's unique capability for dynamic evaluation was described by Chun and Cho, who demonstrated ≤2mm of proximal tendon excursion during elbow range of motion as a marker of stable fixation [14]. Furthermore, Schoch et al. established that consistent visualization of the LHBT along its entire course, from the articular margin to the subpectoral tenodesis site in both transverse and longitudinal planes, serves as a validated criterion for intact tenodesis (positive predictive value of 93%) [11]. 

The failure of interference screw fixation, as observed in this case, is a rare but significant complication of biceps tenodesis. Ultrasound's ability to detect such complications early, without the need for more expensive and time-consuming imaging modalities like MRI or CT, underscores its value in postoperative follow-up. Additionally, ultrasound's portability and lack of radiation burden (unlike CT scan) make it an attractive option for both clinicians and patients. 

Posterior acoustic enhancement was seen post-tenodesis in the presence of an absent LHBT. Enhancement is typically associated with fluid-filled or cystic structures; in this case, it may suggest a biceps tendon sheath effusion due to the known communication between the bicipital groove and the glenohumeral joint. Alternatively, this appearance could represent an artifact, potentially influenced by machine settings such as time-gain compensation. Similar enhancement has been described in the setting of rotator cuff pathology, including full-thickness supraspinatus tears [15]. In contrast, posterior acoustic shadowing, more commonly seen in shoulder ultrasound, is typically associated with dense structures such as calcific deposits, as in calcific tendinopathy [16]. The unexpected presence of enhancement rather than shadowing in this case raises considerations of the synovial fluid presence or a technical artifact, meriting further exploration in future studies to identify and validate post-tenodesis sonographic characteristics. 

These modality-specific strengths support the use of both imaging techniques in follow-up care. While MRI remains superior for anatomical detail, ultrasound offers a dynamic, accessible, and cost-effective alternative that enhances early complication detection and may reduce MRI referrals when used appropriately in a resource-limited situation. The rapid turnaround of ultrasound evaluations directly benefits both clinicians (by optimizing imaging workflows) and patients (through faster results and shorter wait times), making a strong case for its inclusion in routine postoperative follow-up. The challenge lies in acquiring consistent imaging and interpretation in musculoskeletal ultrasound as it is skill-dependent and requires dedicated training and clinical exposure to maximize diagnostic effectiveness.

Future research should focus on standardized characterization of post-tenodesis ultrasound findings, particularly the appearance of the bicep groove which had shown posterior acoustic enhancement rather than the expected shadowing observed in our case. Such investigations would further clarify the observations made in this case. 

## Conclusions

This case highlights the role of ultrasound as a promising, accessible, and cost-effective imaging modality for postoperative monitoring of LHBT tenodesis. It demonstrated a strong correlation with MRI in assessing tendon integrity, implant positioning, and potential complications, supporting its utility in routine orthopedic and sports medicine practice. Despite some limitations, such as operator dependence and reduced sensitivity for partial-thickness tears, ultrasound remains especially useful in resource-limited settings. Further research, including prospective studies and case series, is needed to develop standardized protocols, validate potential sonographic markers like posterior acoustic enhancement, and compare long-term cost-effectiveness with MRI. Overall, ultrasound is increasingly positioned as a practical and essential tool for postoperative monitoring in LHBT tenodesis.
